# A Manual of Bandaging—Adapted for Self Instruction

**Published:** 1895-04

**Authors:** 


A MANUAL OF BANDAGING—ADAPTED FOR SELF-INSTRUCTION. By C. Henri Leonard, A. M., M. D., Professor of the Medical and Surgical Diseases of Women, and Clinical Gynæcology Detroit College of Medicine; Member of American Medical Association, etc. With One Hundred and Thirty-nine engravings. Sixth Edition. Price, $1.50. Detroit: The Illustrated Medical Journal Co. 1895.

Besides a clear description of the bandages to be applied to all parts of the body, illustrations are profuse. The direction of each turn of the bandage is shown with arrowheads and each turn numbered, making the illustrations very plain and easy to follow. Roller bandages, Ts, Cravats, Slings, Tailed, Adhesive and Plaster bandages and immovable dressings are given; also strapping, compressing and poulticing, with method of making and applying the same.

The book is small, nicely bound, splendidly illustrated.

